# Size-Based Enrichment of Exfoliated Tumor Cells in Urine Increases the Sensitivity for DNA-Based Detection of Bladder Cancer

**DOI:** 10.1371/journal.pone.0094023

**Published:** 2014-04-14

**Authors:** Elin Andersson, Kenneth Steven, Per Guldberg

**Affiliations:** 1 Danish Cancer Society Research Center, Copenhagen, Denmark; 2 Department of Urology, Copenhagen University Hospital, Herlev, Denmark; National Cancer Institute, National Institutes of Health, United States of America

## Abstract

Bladder cancer is diagnosed by cystoscopy, a costly and invasive procedure that is associated with patient discomfort. Analysis of tumor-specific markers in DNA from sediments of voided urine has the potential for non-invasive detection of bladder cancer; however, the sensitivity is limited by low fractions and small numbers of tumor cells exfoliated into the urine from low-grade tumors. The purpose of this study was to improve the sensitivity for non-invasive detection of bladder cancer by size-based capture and enrichment of tumor cells in urine. In a split-sample set-up, urine from a consecutive series of patients with primary or recurrent bladder tumors (N = 189) was processed by microfiltration using a membrane filter with a defined pore-size, and sedimentation by centrifugation, respectively. DNA from the samples was analyzed for seven bladder tumor-associated methylation markers using MethyLight and pyrosequencing assays. The fraction of tumor-derived DNA was higher in the filter samples than in the corresponding sediments for all markers (*p*<0.000001). Across all tumor stages, the number of cases positive for one or more markers was 87% in filter samples compared to 80% in the corresponding sediments. The largest increase in sensitivity was achieved in low-grade Ta tumors, with 82 out of 98 cases positive in the filter samples (84%) versus 74 out of 98 in the sediments (75%). Our results show that pre-analytic processing of voided urine by size-based filtration can increase the sensitivity for DNA-based detection of bladder cancer.

## Introduction

Bladder cancer is the seventh most common cancer worldwide and accounts for more than 150,000 deaths each year [1]. More than 90% of bladder tumors are transitional cell carcinomas (also called urothelial carcinoma) arising from the urothelial cells lining the bladder. Typical symptoms of bladder cancer are microscopic and macroscopic hematuria, painful urination and polyuria. However, none of these symptoms is specific for the disease and can be caused by a range of other conditions including cystitis, kidney stones and prostate disease. The gold standard for diagnosing bladder cancer is transurethral resection of the bladder tumor (TURBT). Most patients are diagnosed with non-invasive bladder cancer (stage Ta), which has a five-year survival rate of 90%. However, the recurrence rate is high (50–70%) and 10–25% progress to invasive bladder cancer, warranting long-term follow-up by cystoscopy in patients with non-invasive tumors [2]–[4]. Cystoscopy is an invasive method that is uncomfortable for patients and requires great technical and financial resources. It is, therefore, important to develop non-invasive methods that are simple and cost-effective for diagnosis and follow-up of bladder cancer.

Voided urine specimens from patients with bladder tumors usually contain exfoliated tumor cells that may be detected by cytological analysis. Urine cytology is a non-invasive method that has a high specificity but a low sensitivity (<40%), especially in patients with low-grade tumors [5], [6]. A more sensitive non-invasive method for detection of bladder cancer is based on analysis of tumor-specific alterations in DNA isolated from urine sediments. Chromosomal losses and somatic mutations in specific genes such as *FGFR3* and *TP53* have all been used successfully as biomarkers for detection of various stages of bladder cancer [7]–[9]. In recent years, aberrant hypermethylation at promoter CpG islands has been shown to occur frequently and early in cancer development and may even precede genetic alterations such as mutations and genomic rearrangement in cancerogenesis [10]. Several studies have reported hypermethylation of promoter CpG islands in bladder tumors (reviewed in Refs. [11]–[13]) and these changes can be detected in urine sediments from bladder cancer patients [14], [15]. There is no single DNA-methylation marker that defines all types of bladder tumor, and most studies have utilized a panel of markers to detect bladder cancer in clinical samples. The sensitivity and specificity of DNA-based bladder tumor detection vary considerably across studies, which may be attributed to choice of markers and methods for assessing these markers, as well as differences in the representation of the various tumor stages in patient cohorts [16]–[23].

In studies where paired tumor samples and sediments from urine have been analyzed in parallel for the same panel of DNA markers, the sensitivity of detection is consistently lower in urine [15], [17], [20]. Exfoliation of tumor cells into the urine depends on tumor characteristics such as size, stage and grade and also shows great intra-individual variation [24]. Especially small non-invasive tumors are less likely to shed enough cells into urine to be detected in subsequent analysis. In addition, urine from bladder cancer patients may contain an increased number of other cell types, including white blood cells, which can impact the sensitivity of sediment analysis. Therefore, a method that allows for an enrichment of tumor cells in urine specimens may increase the robustness and sensitivity for non-invasive detection of bladder cancer.

Tumor cells derived from epithelial cells are generally larger than white blood cells, and this size difference could potentially be exploited to enrich for tumor cells in heterogeneous biological samples such as urine. Previous studies have used filters for size-based isolation of rare circulating tumor cells (CTCs) in peripheral blood [25], [26]. The idea of capturing cells in urine on a filter was introduced more than thirty years ago [27], and later studies have utilized membrane filters for preparation of epithelial cells from urine for detection of urothelial carcinoma by cytology or fluorescence in situ hybridization (FISH) analysis [28]–[30]. In this study, we have tested a simple and cost-effective procedure for pre-analytic urine filtration to increase the fraction of tumor cells and thus the sensitivity for DNA-based detection over unfiltered sediments. In a split-sample set-up, urine samples from bladder cancer patients were assessed for the presence of tumor DNA with a panel of methylation markers frequently detected in bladder cancer. The fractions of methylated alleles were quantified in sedimented and filtered samples by MethyLight assays and pyrosequencing. We found that this filtering method increased the fraction of tumor-derived DNA and also improved the sensitivity and robustness of bladder tumor detection from urine samples.

## Materials and Methods

### Ethics Statement

The study was approved by The Copenhagen Ethical Committee, and written informed consent was obtained from all patients and controls at inclusion.

### Collection of Urine Samples

Voided morning urine samples were collected from bladder cancer patients admitted for TURBT at Copenhagen University Hospital, Herlev, Denmark, between June 2010 and October 2011, and from healthy volunteers without known urological malignancies. Samples were sent to the Danish Cancer Society and processed within 4–6 hours after voiding.

### Processing of Urine Samples

Fifty milliliters of each urine sample were sedimented by centrifugation at 2,000 g for 10 min. The pellet was washed in phosphate-buffered saline (PBS) followed by another 10 min centrifugation. The supernatant was discarded and the pellet was resuspended in approximately 200 μl of PBS. In parallel, urine from the same sample was drawn into a disposable syringe and passed through a Whatman Nuclepore track-etched polycarbonate hydrophilic membrane filter (diameter 25 mm) mounted in the corresponding filter holder (Whatman, Maidstone, UK). The urine sample was passed through the filter by positive force until a resistance was felt (saturation), with a maximum of 125 ml. All filters were rinsed with PBS before removal from the filter holder. Urine sediments and filters with filter content were stored at −80°C until further processing.

### DNA Isolation and Bisulfite Conversion

DNA was isolated from urine sediment and filter samples using the Qiagen Mini Prep kit (Qiagen GmbH, Hilden, Germany) according to manufacturer’s instructions. Filter samples and sediments were incubated with ATL buffer and proteinase K at 56°C for at least one 1 hour or overnight. Subsequent processing was done according to the protocol for DNA purification from tissues. DNA from filters and sediments was eluted in 50 μl and 100 μl of buffer AE, respectively, and stored at −80°C. The DNA concentration was measured using a NanoDrop spectrophotometer (Thermo Scientific, Wilmington, DE, USA).

Bisulfite conversion was done using the EZ DNA Methylation-Gold Kit (Zymo Research Corp, Orange, CA, USA) according to the manufacturer’s protocol. The bisulfite-converted DNA was eluted in 2×10 μl of M-Elution Buffer and stored at −80°C. For paired samples (sediment and filter), the same amount of DNA was used, with a maximum of 500 ng. In cases where the DNA concentration was too low to be accurately determined using the NanoDrop spectrophotometer, the maximum sample volume (20 μl) was used. Normal human bladder epithelium derived from a 66 years old male was purchased from Capital Biosciences (Rockville, MD, USA).

### Cell Culture

The T24 cell line (DSMZ, Braunschweig, Germany) was cultured in DMEM medium supplemented with 10% fetal bovine serum. Lymphocytes were isolated from blood from a healthy donor essentially as described [31] and stored at −80°C until use. Cells in single-cell suspension were counted and measured using a Countess Automated Cell Counter (Invitrogen, Carlsbad, CA, USA). The two cell types were mixed at the indicated ratios and processed by centrifugation and filtration as described above for urine samples.

### Denaturing Gradient Gel Electrophoresis

Mutation analysis of *HRAS* exon 2 by denaturing gradient gel electrophoresis (DGGE) was done essentially as described [32]. Primer sequences and PCR conditions are listed in Table S1. PCR products were loaded onto a 0% denaturant/6% polyacrylamide-90% denaturant/9% polyacrylamide double-gradient gel [33]. The gels were run at 170 V for 4.5 h in TAE buffer kept at a constant temperature of 58°C, stained with ethidium bromide and photographed under UV transillumination.

### MethyLight

Real-time quantitative methylation-specific PCR (MethyLight; Ref. [34]) was performed essentially as described [20]. Primer and probe sequences are listed in Table S1. Reactions were performed on the LightCycler 480 platform using the LightCycler 480 Probes Master Kit (Roche, Mannheim, Germany) and 1 μl of bisulfite-treated DNA per reaction. The specificity of each assay was established using *in vitro* methylated DNA (IVM; CpGenomeTM Universal Methylated DNA, Chemicon/Millipore, Billerica, MA) and DNA from selected cancer cell lines as positive and negative controls for methylation, respectively, and water and non-bisulfite treated genomic DNA as negative controls for amplification. A MethyLight assay for *ALUC4* and a dilution series of IVM were used to determine the DNA concentration of the sample after bisulfite treatment [20], [35]. Cases were discarded if either of the paired sediment or filter samples had a concentration below the equivalent of 0.25 ng/μl non bisulfite-treated DNA. Methylation levels were calculated as percent methylated reference (PMR; Ref. [35]) by normalizing marker-specific reaction values to *ALUC4* values relative to the same values for fully methylated control (IVM). The small quantities of starting DNA for many of the samples limited the number of markers that could be tested, and all analyses were performed as single reactions. The background methylation level for each marker was determined using DNA from sedimented and filtered urine samples from 11 healthy controls as well as Human Genomic DNA (Roche, Mannheim, Germany). The cut-off PMR values were 3 for *HOXA9*, 2 for *POU4F2*, 0.5 for *SALL3* and 2 for *VIM2.*

### Pyrosequencing

Pyrosequencing assays for the *HRAS* p.G12V (c.35G>T) mutation and *BCL2* promoter methylation were designed using the PyroMark assay design software (Qiagen GmbH, Hilden, Germany). Primer sequences and PCR conditions are listed in Table S1. PCR was carried out in a final volume of 25 μl containing PCR buffer (Qiagen GmbH, Hilden, Germany), 200 μM each dNTP, 0.4 μM each primer and 1 U of Taq HotStarTaq DNA Polymerase (Qiagen GmbH, Hilden, Germany). Pyrosequencing was performed on a PyroMark Q24 platform, using PyroMark Gold Q24 Reagents (Qiagen GmbH, Hilden, Germany). Analysis of the results was carried out with the PyroMark Q24 software (Qiagen GmbH, Hilden, Germany). Average methylation levels for *BCL2* were calculated for the seven CpG sites included in the assay. The background signal for the *BCL2* methylation assay was set at 5% based on the analysis of bisulfite-treated Human Genomic DNA (Roche, Mannheim, Germany). For the *BCL2* methylation analysis, only clinical samples with a concentration equivalent to 5 ng/μl non bisulfite-treated DNA or more were included.

## Results

### Capture and Enrichment of Bladder Tumor Cells on a Membrane Filter

**Figure 1:**
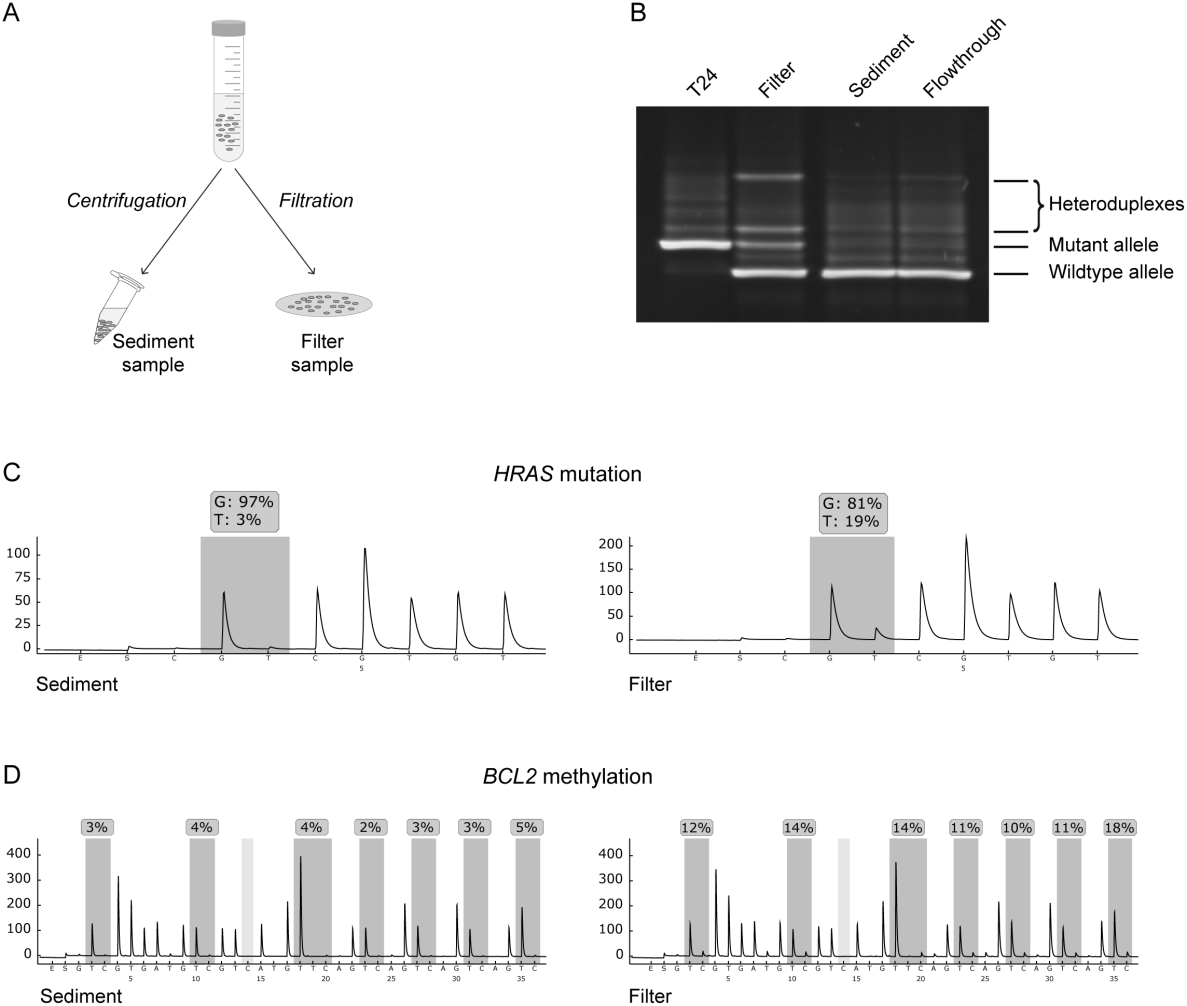
Filter-based capture and enrichment of bladder cancer cells. (**A**) Schematic drawing of the split-sample experimental set-up. Urine sample or cell suspension is divided in two and subjected to centrifugation and filtration, respectively. DNA from cells in sediment or captured on filter is then isolated and analyzed for tumor-specific markers. (**B**) Detection of the *HRAS* p.G12V mutation by DGGE. The cell line T24 is homozygous for the mutant allele. Sediment and flowthrough samples display only the wildtype allele, while the filter sample shows both mutant and wildtype alleles. Heteroduplexes are hybrid molecules made up of one mutant strand and one wildtype strand. (**C**) Pyrographs showing the distribution between wildtype (G) and mutant (T) *HRAS* alleles in the sediment and filter samples. (**D**) Pyrographs of *BCL2* promoter CpG island methylation analysis in the sediment and filter samples. The individual CpG sites in the target region are indicated by dark grey shading, and the percentage of methylated alleles (C) is indicated at each site.

To test the ability of membrane filters to capture bladder tumor cells, we first used a model system designed to resemble urine specimens containing low fractions of tumor cells. One experiment is shown in Figure 1. Purified cultured peripheral blood lymphocytes (diameter 7–8 μm) were spiked with 0.5% T24 bladder cancer cells (diameter 16–17 μm). Part of this cell mixture was sedimented by centrifugation, while the remainder was passed through a membrane filter with a pore size of 8 μm (Figure 1A). The flowthrough from the filter was also collected and sedimented by centrifugation. To assess if filtration increases the fraction of tumor cells in the sample, DNA was isolated and analyzed for two tumor-specific alterations present in T24 cells; the *HRAS* p.G12V mutation and hypermethylation of the *BCL2* promoter. Figure 1B shows the physical resolution of mutant and wild-type *HRAS* alleles using DGGE, which has a detection level of approximately 2% [36]. The mutant *HRAS* allele was clearly detectable as homo- and heteroduplexes in the filter sample but not in the sediment or flowthrough samples (Figure 1B). Quantitative analysis of the same samples using pyrosequencing showed an increase in the ratio of mutant (T) over wildtype (G) *HRAS* alleles from 3–4% in the sediment and flowthrough samples to 19% in the filter sample (Figure 1C and data not shown). Bisulfite pyrosequencing showed a similar increase in the fraction of *BCL2* hypermethylated alleles, specific for T24 cells, upon filtration (Figure 1D).

**Figure 2:**
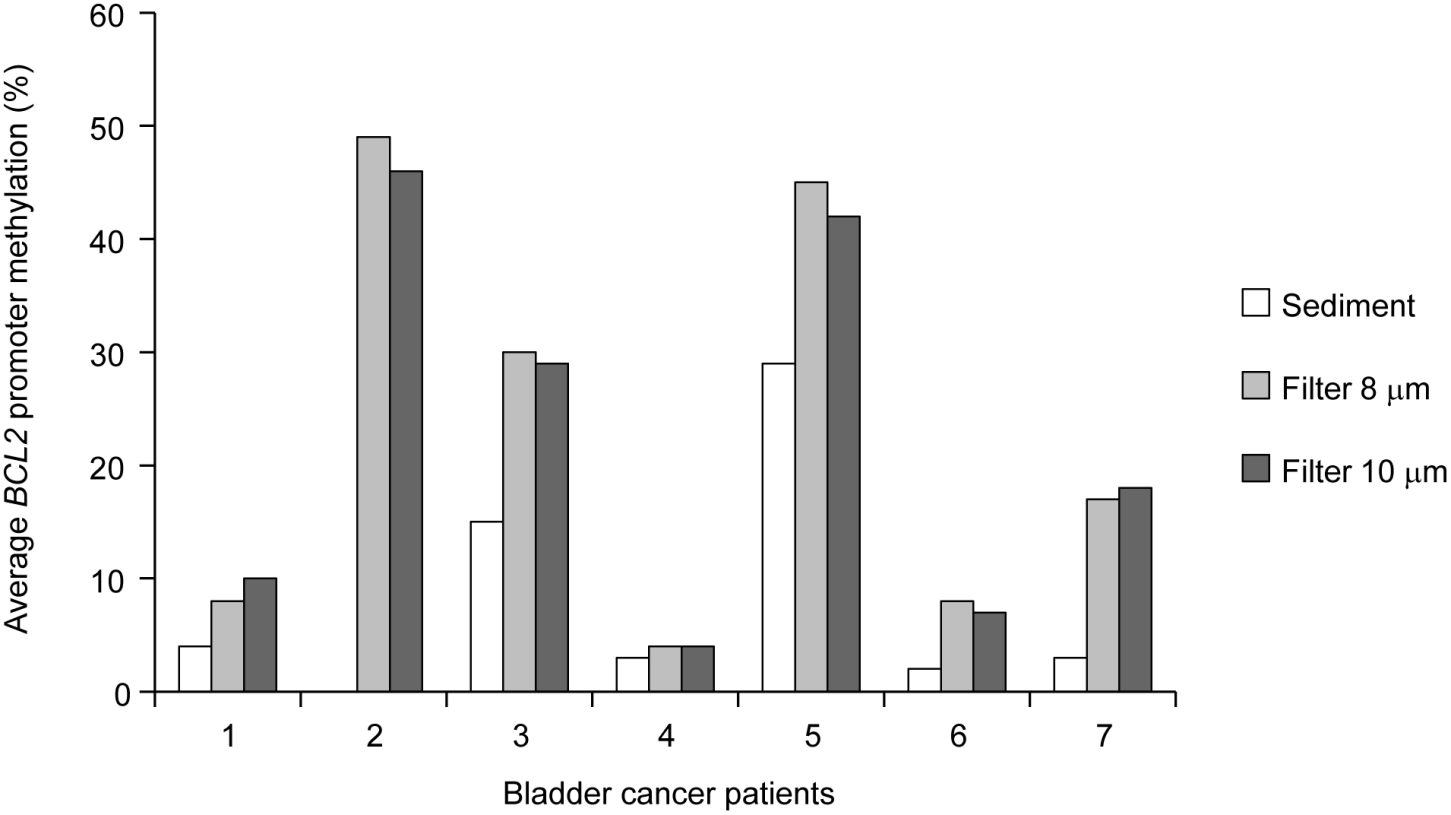
Comparison of levels of tumor-derived DNA in urine samples prepared by sedimentation or filtration. Average methylation levels of the *BCL2* promoter CpG island in urine samples from seven bladder cancer patients, as determined by pyrosequencing. Sediment and two filter samples (8 and 10 μm pore size, respectively) were prepared from each urine sample. The sediment sample from patient 2 is missing due to failed DNA extraction.

To test the filtration method in a clinical setting, urine samples were obtained from 15 bladder cancer patients admitted for TURBT. In this series of samples, we also assessed whether the pore size could impact the ratio of tumor cells to normal cells. From each urine sample, 50 ml were prepared by centrifugation, while the remaining volume was divided equally and passed through two filters with pore sizes of 8 μm and 10 μm, respectively. DNA was isolated from all urine sediments and filter samples and examined for *BCL2* methylation using a highly specific and sensitive MethyLight assay. Seven out of the 15 urine samples were positive for this marker, consistent with previous reports showing *BCL2* hypermethylation in a large percentage of bladder tumors [16], [22]. To estimate the fraction of tumor-derived DNA, methylation levels were calculated for the *BCL2*-positive samples using normalized values (PMR). There was only a small difference in methylation levels between the 8 and 10 μm filters, with slightly higher levels in the 8 μm filter (Table S2). Quantitative analysis of *BCL2* methylation by pyrosequencing confirmed the results of the MethyLight analysis (Figure 2). Notably, all filter samples (both 8 μm and 10 μm) showed higher *BCL2* methylation levels than the corresponding sediment samples, indicating a higher fraction of tumor cells.

### Urine Filtration Increases the Fraction of Tumor DNA in Clinical Samples

Having obtained proof of principle that membrane filters can capture and enrich for bladder tumor cells in urine samples, we carried out a clinical study of 220 consecutive bladder tumor patients. Demographic and clinico-pathological characteristics of these patients are listed in Table S3. There was an equal number of patients with primary and recurrent tumors in this cohort. Morning urine samples were collected and processed according to the split-sample design illustrated in Figure 1A, using an 8 μm filter. DNA was isolated from all urine sediments and filter samples and treated with sodium bisulfite. The concentration of bisulfite-treated DNA in each sample was determined using a MethyLight assay for *ALUC4*. Thirty-one paired samples were discarded because of low DNA yield (Table S3).

**Figure 3:**
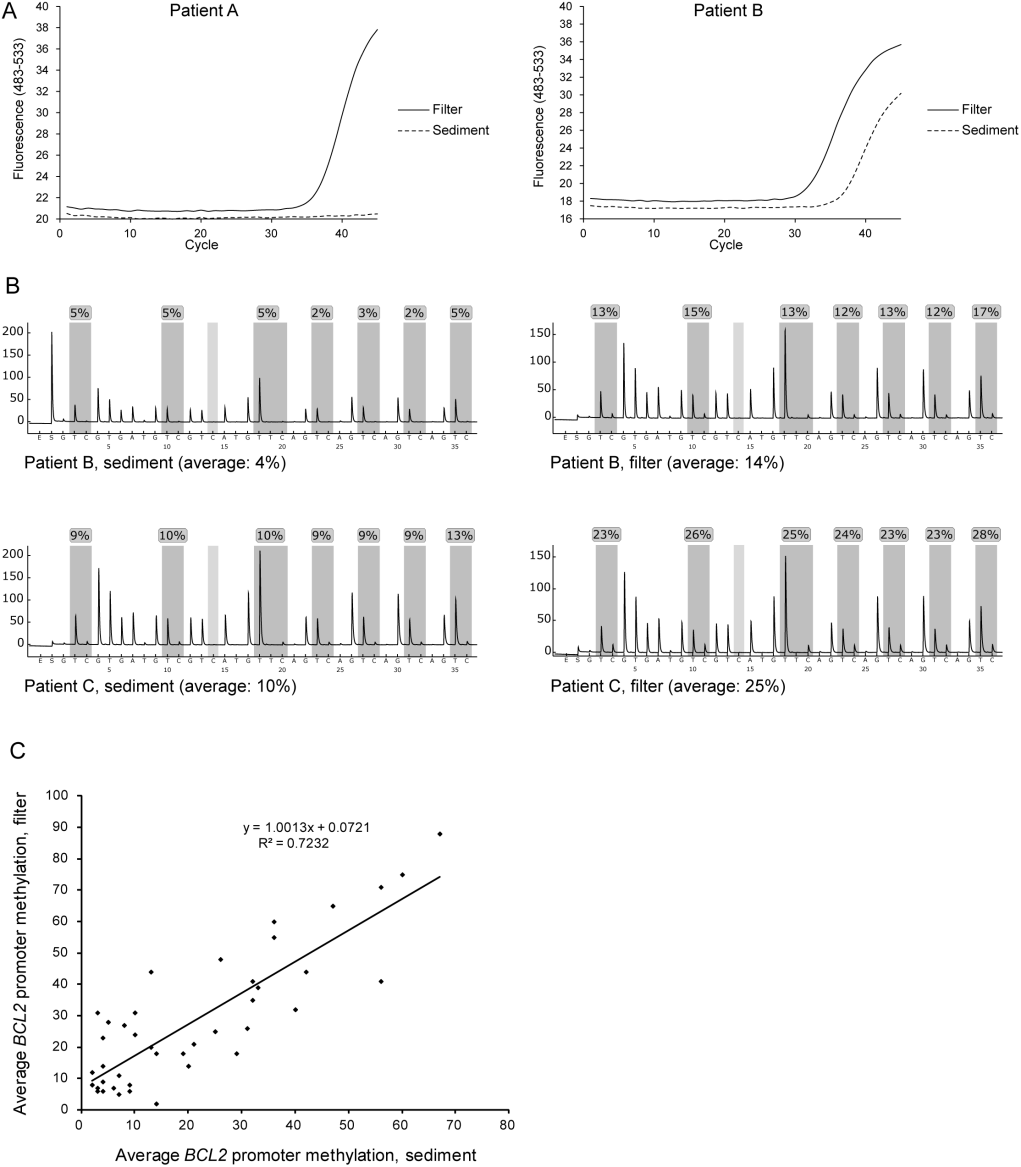
Analysis of tumor-derived DNA in paired urine samples (sediment and filter) from bladder cancer patients. (**A**) Examples of MethyLight analysis of *BCL2* promoter CpG island methylation. In patient A, amplification is seen in the filter sample but not in the sediment sample. In patient B, amplification is seen in both samples; however with a higher Ct value for the sediment sample. (**B**) Examples of *BCL2* promoter CpG island methylation analysis by pyrosequencing in paired samples. Average methylation level is calculated for the seven individual CpG sites assayed (indicated by dark grey shading). The average methylation level in the sediment sample from patient B was below the background signal for pyrosequencing, in contrast to the MethyLight assay (**A**), showing the difference in analytical sensitivity between the two assays. (**C**) Average methylation levels for paired samples as determined by pyrosequencing. Shown are the results for cases where at least one of the paired samples showed signals above the background for pyrosequencing (N = 42).

**Figure 4:**
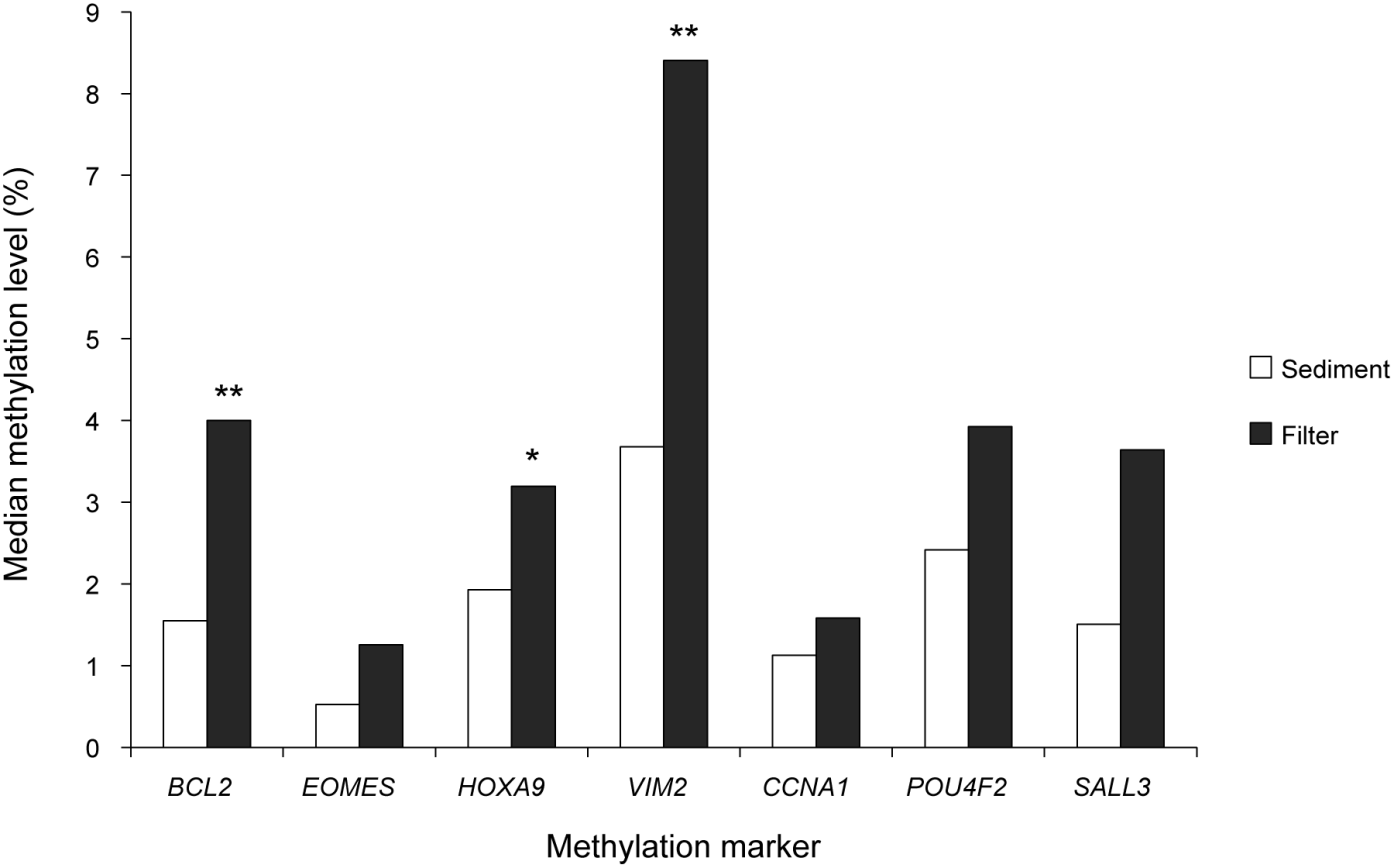
Levels of DNA methylation in paired urine samples (sediment and filter) from patients with bladder cancer (N = 189). Shown is the median of normalized values (percent methylated reference, PMR) from MethyLight analysis of seven methylation markers. For each marker, the methylation levels ranged from <1% to 100% (not shown). *, p<0.05; **, p<0.01.

Screening of all 189 paired samples for *BCL2* methylation by MethyLight analysis identified 121 cases positive for this marker (examples are shown in Figure 3A). In 60 of these cases, both filter and sediment samples contained sufficient amounts of DNA for reliable quantitative analysis by pyrosequencing (Figure 3B). In 18 out of 60 cases, the average *BCL2* methylation level was below the background signal for pyrosequencing in both sediment and filter samples. Of the remaining 42 cases, 29 (69%) showed higher methylation levels in the filter sample compared with the sediment sample, five cases (12%) showed equal methylation levels (<2 percent points difference), and eight cases (19%) showed higher methylation levels in the sediments than in the corresponding filters (Figure 3C). The average methylation level was 28% in filter samples compared to 21% in sediments (*p*<0.001, paired t-test). The increase in methylation levels from sediment to filter ranged from 3 to 35 percent points, with a median value of 10 percent points. Quantitative analysis of all 121 cases positive for *BCL2* methylation using normalized values (PMR) from the MethyLight analysis showed an average methylation level of 10.0% in the sediment samples and 14.4% in the filter samples, with the median level increasing from 1.6% to 4.0% (Figure 4; *p*<0.001, paired t-test).

To analyze samples negative for *BCL2* methylation, we expanded the MethyLight-based analysis by six additional promoter CpG islands previously reported to be frequently hypermethylated in bladder cancer, including *CCNA1*, *EOMES*, *HOXA9*, *POU4F2*, *SALL3* and *VIM2* [16], [18], [23]. We have validated these markers together with *BCL2* in a panel of 51 bladder tumors representing various tumor stages (22 low grade Ta tumors, 2 high grade Ta tumors, 8 T1 tumors, 17≥T2 tumors, and 2 Tis). Each of these markers was positive in >45% of the tumors in this panel, and 48 of the tumors (94%) were positive for at least one marker. Tumor specificity of the markers was confirmed by analysis of normal bladder tissue.

Quantitative analysis of all seven DNA methylation markers showed an increase in methylation levels in filter samples compared with sediments (Figure 4; *p*<0.000001, paired t-test). This difference was also statistically significant for some individual markers, including *BCL2*, *HOXA9* and *VIM2* (Figure 4).

### Urine Filtration Increases the Sensitivity for Detection of Bladder Cancer

We finally addressed whether the increased fractions of tumor-derived DNA achieved by urine filtration could impact the sensitivity for detection of bladder cancer. To determine the levels of background methylation for each of the seven markers, we examined DNA from filter and sediment urine samples from 11 healthy controls as well as DNA from peripheral blood lymphocytes. Late-cycle amplification was occasionally observed for four of the markers (*HOXA9*, *POU4F2*, *SALL3* and *VIM2*), some of which were maintained at repeated analysis. On the basis of these data, a cut-off value was defined for each of these markers above which a sample was considered positive. *BCL2, CCNA1* and *EOMES* did not show any background methylation.

**Figure 5:**
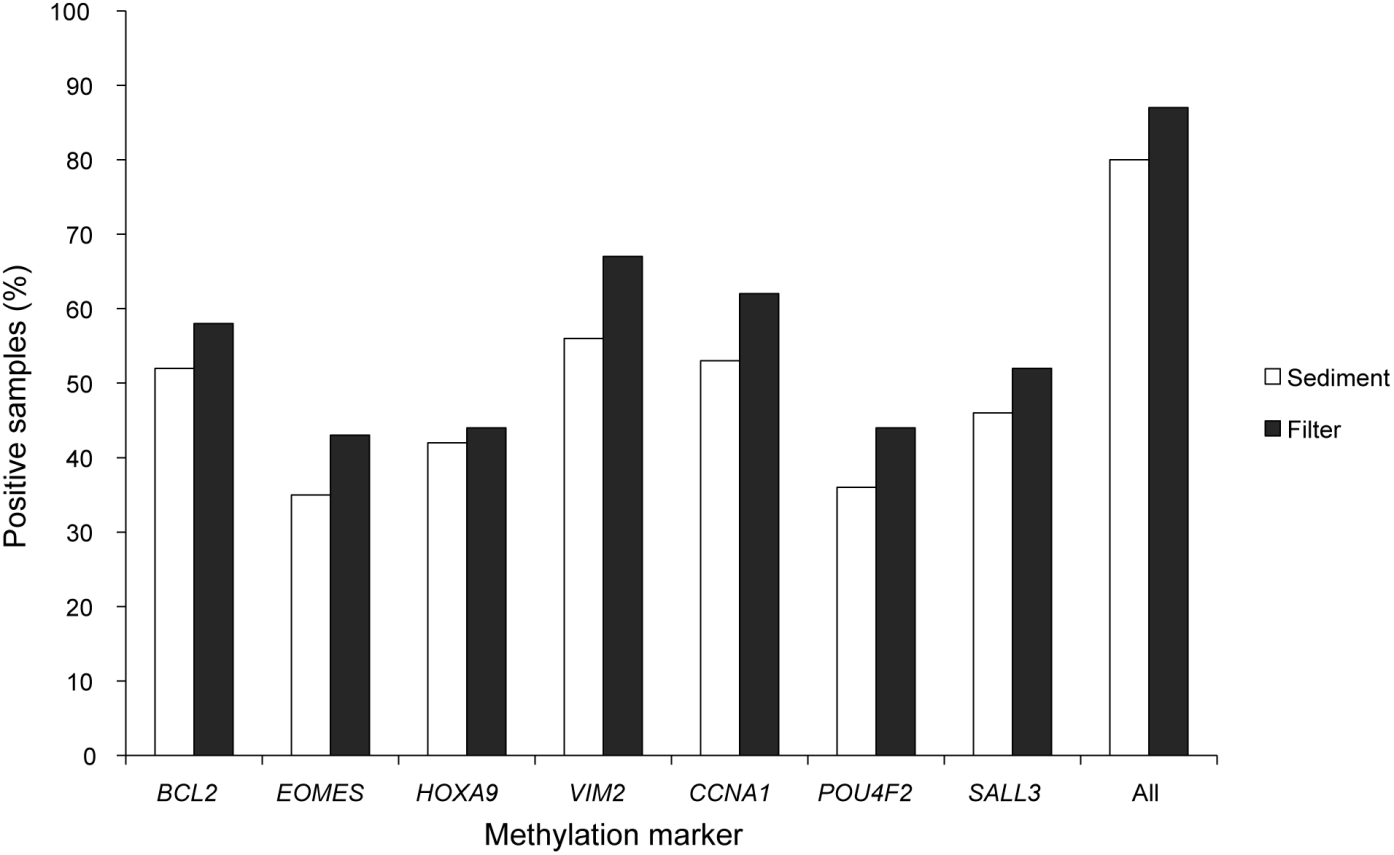
Sensitivity for detection of bladder cancer in paired sediment and filter samples (N = 189). Shown are the percentages samples positive for the seven DNA-methylation markers. The last column (“All”) shows the percentage of samples positive for one or more markers.

With positivity defined as hypermethylation of at least one of the seven markers, the sensitivity across all tumor stages was 80% (152/189) in the urine sediments, while it was 87% (164/189) in the filter samples. This pattern was clear also for individual markers, which all displayed a higher sensitivity in the filter samples (Figure 5). The marker showing the highest sensitivity was *VIM2*, which was positive in 56% of the sediment samples and 67% of the filter samples. The six other markers had sensitivities of 35–53% in sediments and 43–62% in filters. The sensitivity for higher-stage tumors (≥T1) was generally high (>90%) in sediments and was only slightly, albeit consistently, increased after filtration. Notably, however, a dramatic effect was seen for low-grade Ta tumors, where the sensitivity increased from 75% (74/98) in sediments to 84% (82/98) in filters (Table 1). Among the 95 primary tumors, 80 (84%) were detected in the sediment and 84 (88%) were detected in the filter. Among the 94 recurrent tumors, 73 (78%) were detected in the sediment and 80 (85%) were detected in the filter.

**Table 1:** Sensitivity of seven DNA methylation markers in filter and sediment samples from bladder cancer patients (N = 189).

Pathology	Sediment	Filter
All	152/189 (80%)	164/189 (87%)
Low-grade Ta/dysplasia	74/98 (75%)	82/98 (84%)
High-grade Ta	24/31 (77%)	25/31 (81%)
T1	27/30 (90%)	28/30 (93%)
>T2	17/19 (89%)	18/19 (95%)
CIS	24/26 (92%)	25/26 (96%)

Positivity was defined as hypermethylation of one or more markers.

## Discussion

Non-invasive assays that can accurately and reliably detect bladder cancer will have a substantial impact on patients and the healthcare system by reducing the need for frequent, costly and uncomfortable cystoscopy. Significant progress has been made in identifying urine-based biomarkers that can outperform urine cytology, and some of these markers have been developed into commercial tests. Nevertheless, challenges remain in reaching the sensitivity of cystoscopy [37]–[39]. The goal of this study was to increase the sensitivity for DNA-based detection of bladder cancer in urine samples through a simple filtration procedure, to enrich for tumor cells. In a large consecutive cohort of bladder tumor patients, we have tested a commercial track-etched polycarbonate filter with a pore size of 8 μm and compared it with standard urine sediment analysis. Quantitative analysis across a panel of seven DNA methylation markers showed significantly increased levels of tumor DNA in filtered urine samples compared to the corresponding sediments, suggesting that the filter captures bladder tumor cells preferentially over other cells present in urine. Most important, the filtration procedure identified a greater number of samples from bladder tumor patients as positive, resulting in an overall diagnostic sensitivity of 87% in filter samples compared with a sensitivity of 80% in the corresponding sediments.

Low-grade, low-stage bladder tumors represent the greatest challenge in urine-based detection approaches, including standard cytology and FISH, as these tumors tend to shed lower numbers of cells into the urine [40], [41]. This limitation was also evident in our DNA-based approach, where the sensitivity in urine sediments was close to or above 90% for stage ≥T1 tumors, while it was only 75% for low-grade Ta tumors. Interestingly, filtration of the urine specimens increased the sensitivity for low-grade Ta tumors to 84%, suggesting that this procedure may alleviate some of the difficulties in detecting these tumors. Among the total of 189 patients investigated, 16 cases were negative for all methylation markers in both filter and sediment samples, and 13 of these had high- or low-grade Ta tumors. We [20] and others [42] have previously shown that a subset of superficial bladder tumors display low rates of hypermethylation events. Interestingly, these tumors instead show relatively high rates of activating *FGFR3* mutations [20] and, therefore, the combination of methylation markers and *FGFR3* mutations may increase the sensitivity for detection of superficial tumors in a diagnostic setting [20], [43]. We restricted our analysis to one type of marker (DNA hypermethylation) to provide a consistent and comparable quantitative measure for evaluating the two procedures for preparation of diagnostic cells (filtration and sedimentation), which was the main purpose of our study. Furthermore, we discarded a relatively large number of samples (14%) due to low DNA yield, which could compromise the quantitative analysis. However, these samples could well have been qualitatively tested for DNA methylation markers in a diagnostic setting.

As up to 70% of patients with non-invasive bladder cancer will experience relapse, non-invasive urine tests are highly desirable for recurrence surveillance. Half of the patients in our cohort presented with a recurrent tumor, and in this group, as for patients with primary tumors, urine filtration provided a higher sensitivity than sedimentation (85% vs. 78%). These data suggest that urine filtration may also improve diagnosis of bladder cancer recurrence, which may be particularly useful as recurrent tumors often are smaller and shed fewer cells than primary tumors. Nevertheless, this procedure may not alleviate other limitations of DNA methylation-based recurrence surveillance, including the high positive rate among cystoscopy-negative cases, which may be caused by epigenetic changes in normal-appearing urothelium (epigenetic field defect) [44], [45].

An obvious critical parameter for size-based enrichment of tumor cells in biological specimens is the pore size of the filter. Ideally, the pores should be large enough to exclude normal cells and small enough to retain tumor cells, taking into account the deformability of cells under pressure. Previous studies aiming at enriching rare CTCs in blood by filtration found that a 8 μm pore size filter depleted samples of 99.9% of leukocytes while retaining 85–100% of carcinoma cells [25], [46]. With a larger pore size (12–14 μm), fewer leukocytes but also considerably fewer carcinoma cells (down to 18%) were retained on the filter [46]. Using a commercial track-etched polycarbonate filter with a pore size of 8 μm, which is similar to the filters used in these previous studies, we were able to enrich the fraction of tumor cells in urine samples. However, we rarely obtained pure tumor cell preparations after filtration, and this problem was not alleviated by increasing the pore size to 10 μm. In this context, it is important to consider that size-based enrichment of carcinoma cells in urine presents a greater challenge than filtration of blood due to the greater complexity of urine in terms of cellular composition. Urine from healthy individuals contains a variety of cell types, including cells of hematologic and epithelial origin, and the morphology, distribution and absolute numbers of these cells can change dramatically in different pathological states. The presence of normal epithelial cells shed from the lining of the bladder represents a particular problem for size-based enrichment procedures as these cells are generally large (20–100 μm) and overlap in size with carcinoma cells. The wildtype signals consistently seen in samples after filtration may originate from such large normal cells.

In conclusion, we have shown that filtration of voided urine from patients with bladder cancer can capture and increase the fraction of tumor cells, providing a simple and versatile means for improving the accuracy and sensitivity for non-invasive detection of this cancer. As the filtration procedure requires only a filter, a filter holder and a syringe and can be performed with little training, it may provide a cost-equivalent alternative to urine sedimentation and will allow on-site preparation of diagnostic cells which can then be shipped to diagnostic laboratories. Although focus in this study was on bladder cancer, the same approach may be applied to other genitourinary cancers where tumor cells are shed into urine, including prostate and upper urinary tract tumors [11].

## Supporting Information

Table S1 Oligonucleotides and amplification conditions.
(DOC)

Table S2 Methylation levels of the *BCL2* promoter CpG island in urine samples from seven bladder cancer patients.
(DOC)

Table S3 Demographic and clinico-pathological characteristics of bladder tumor patients.
(DOC)
