# Supplementary material for: Size-Based Enrichment of Exfoliated Tumor Cells in Urine Increases the Sensitivity for DNA-Based Detection of Bladder Cancer
Source: PLoS One. 2014 Apr 14;9(4):e94023. doi: 10.1371/journal.pone.0094023 (PMC3986060; doi:10.1371/journal.pone.0094023)
Supplement: Table S1 — Oligonucleotides and amplification conditions. (DOC) [file pone.0094023.s001.doc]

| *DGGE* | |  |  |  |  |  |
| --- | --- | --- | --- | --- | --- | --- |
| *Gene* | Fwd primer (5'-3') | Rev primer (5'-3') |  | Amplicon length (bp) | Annealing temp. (°C) | Reference |
| *HRAS* | AGGAGCGATGACGGAATATAAG | [50GC]-GCTCACCTCTATAGTGGGGTCG |  | 174 | 58 | (1) |
|  |  |  |  |  |  |  |
| *Pyrosequencing* | |  |  |  |  |  |
| Gene | Fwd primer (5'-3') | Rev primer (5'-3') | Seq primer (5'-3') | Amplicon length (bp) | Annealing temp. (°C) | Reference |
| *HRAS* | AGGCCCCTGAGGAGCGAT | [Btn]-CTGGATGGTCAGCGCACTCTT | TGGTGGTGGGCGCCG | 82 | 60 |  |
| *BCL2* | AGGTGTAGTTGGTTGGATATT | [Btn]-ATCTTCTCCTCCCAACCC | GTGGATTATAGGTGGTAT | 221 | 58 |  |
|  |  |  |  |  |  |  |
| *MethyLight* | |  |  |  |  |  |
| Gene | Fwd primer (5'-3') | Rev primer (5'-3') | Probe (5'-3') | Amplicon length (bp) | Annealing temp. (°C) | Reference |
| *BCL2* | TCGTATTTCGGGATTCGGTC | AACTAAACGCAAACCCCGC | [6FAM]-ACGACGCCGAAAACAACCGAAATCTACA-[TAMRA] | 84 | 62 | (2) |
| *CCNA1* | TCGCGGCGAGTTTATTCG | CCGACCGCGACAAACG | [6FAM]-CGTTATGGCGATGCGGTTTCGG-[TAMRA] | 85 | 60 | (3) |
| *EOMES* | GTTTGGCGCGTATTTTTATTC | CTCGCAAAAAAAACTACCGAA | [6FAM]-TACCGCCTCGCGCACTCTCC-[TAMRA] | 145 | 58 |  |
| *HOXA9* | GTAGTTTTATAATTTTCGTGGGTC | AACAAAAACGAATCCACGTAA | [6FAM]-AACCATCACCGTACCCAACGC-[TAMRA] | 105 | 60 |  |
| *POU4F2* | GAGTATAATTCGTCGGTCGC | AATACCTAACTCCGCTTACCG | [6FAM]-ACACCCGAAACTACCCCCGC-[TAMRA] | 82 | 58 |  |
| *SALL3* | GTTCGCGTAGTCGTCGTC | TACTCGAAAACCCCGTCA | [6FAM]-ACGACGCGAAACGACCTAACG-[TAMRA] | 203 | 60 |  |
| *VIM2* | TTCGGGAGTTAGTTCGCGTT | ACCGCCGAACATCCTACGA | [6FAM]-TCGTCGTTTAGGTTATCGT-[MGB] | 108 | 62 | (4) |
| *ALUC4* | GGTTAGGTATAGTGGTTTATATTTGTAATTTTAGTA | ATTAACTAAACTAATCTTAAACTCCTAACCTCA | [6FAM]-CCTACCTTAACCTCCC-[MGB] |  | 64 | (5) |

Table S1. Oligonucleotides and amplification conditions

References

(1) Nedergaard T, Guldberg P, Ralfkiaer E, Zeuthen J. A one-step DGGE scanning method for detection of mutations in the K-, N-, and H-ras oncogenes: mutations at codons 12, 13 and 61 are rare in B- cell non-Hodgkin's lymphoma. Int J Cancer 1997;71:364-9.

(2) Weisenberger DJ, Siegmund KD, Campan M, Young J, Long TI, Faasse MA, Kang GH, Widschwendter M, Weener D, Buchanan D, Koh H, Simms L, et al. CpG island methylator phenotype underlies sporadic microsatellite instability and is tightly associated with BRAF mutation in colorectal cancer. Nat Genet 2006;38:787-93.

(3) Brait M, Begum S, Carvalho AL, Dasgupta S, Vettore AL, Czerniak B, Caballero OL, Westra WH, Sidransky D, Hoque MO. Aberrant promoter methylation of multiple genes during pathogenesis of bladder cancer. Cancer Epidemiol Biomarkers Prev 2008;17:2786-94.

(4) Costa VL, Henrique R, Danielsen SA, Duarte-Pereira S, Eknaes M, Skotheim RI, Rodrigues A, Magalhaes JS, Oliveira J, Lothe RA, Teixeira MR, Jeronimo C, et al. Three epigenetic biomarkers, GDF15, TMEFF2, and VIM, accurately predict bladder cancer from DNA-based analyses of urine samples. Clin Cancer Res 2010;16:5842-51.

(5) Weisenberger DJ, Campan M, Long TI, Kim M, Woods C, Fiala E, Ehrlich M, Laird PW. Analysis of repetitive element DNA methylation by MethyLight. Nucleic Acids Res 2005;33:6823-36.
