# Supplementary material for: Size-Based Enrichment of Exfoliated Tumor Cells in Urine Increases the Sensitivity for DNA-Based Detection of Bladder Cancer
Source: PLoS One. 2014 Apr 14;9(4):e94023. doi: 10.1371/journal.pone.0094023 (PMC3986060; doi:10.1371/journal.pone.0094023)
Supplement: Table S2 — Methylation levels of the BCL2 promoter CpG island in urine samples from seven bladder cancer patients. (DOC) [file pone.0094023.s002.doc]

Table S2. Methylation levels of the *BCL2* promoter CpG island in urine samples from seven bladder cancer patients. Percent methylated reference (PMR) was determined using the MethyLight assay.

|  |  | **PMR (*BCL2*)** | | |
| --- | --- | --- | --- | --- |
| **Patient** | **Pathology** | Sediment | Filter (8 µm) | Filter (10 µm) |
| 1 | Ta, LG and HG | 0.18 | 1.23 | 1.10 |
| 2 | Ta, HG and Tis | n.a.* | 9.11 | 11.78 |
| 3 | Tis and T1b | 1.83 | 8.20 | 6.55 |
| 4 | Ta, HG and Tis | 0.25 | 0.72 | 0.87 |
| 5 | Tis | 12.63 | 25.94 | 12.75 |
| 6 | Ta, LG | 0 | 0.31 | 0.24 |
| 7 | Ta, LG | 0.52 | 6.32 | 6.27 |

LG, low grade; HG, high grade. *, Failed DNA extraction
