# Supplementary material for: Size-Based Enrichment of Exfoliated Tumor Cells in Urine Increases the Sensitivity for DNA-Based Detection of Bladder Cancer
Source: PLoS One. 2014 Apr 14;9(4):e94023. doi: 10.1371/journal.pone.0094023 (PMC3986060; doi:10.1371/journal.pone.0094023)
Supplement: Table S3 — Demographic and clinico-pathological characteristics of bladder tumor patients. (DOC) [file pone.0094023.s003.doc]

Table S3. Demographic and clinico-pathological characteristics of bladder tumor patients.

| Characteristic | All patients  (N = 220) | Patients selected for urine DNA analysis (N=189) |
| --- | --- | --- |
|  |  |  |
| **Age (years)** |  |  |
| Median | 73 | 73 |
| Range | 43-94 | 45-94 |
|  |  |  |
| **Sex** |  |  |
| Male | 163 | 135 |
| Female | 57 | 54 |
|  |  |  |
| **Primary/recurrent tumor** |  |  |
| Primary | 110 | 95 |
| Recurrent | 110 | 94 |
|  |  |  |
| **Tumor stage** |  |  |
| Dysplasia | 5 | 3 |
| Ta | 139 | 118 |
| Tis | 26 | 23 |
| T1 | 30 | 29 |
| T2 or higher | 20 | 16 |
|  |  |  |
